# Crop Seed Phenomics: Focus on Non-Destructive Functional Trait Phenotyping Methods and Applications

**DOI:** 10.3390/plants12051177

**Published:** 2023-03-04

**Authors:** Gokhan Hacisalihoglu, Paul Armstrong

**Affiliations:** 1 Biological Sciences Department, Florida A&M University, Tallahassee, FL 32307, USA; 2 USDA-ARS Center for Grain and Animal Health Research, Manhattan, KS 66502, USA

**Keywords:** seeds, phenomics, NIR, Fourier transform, end-use quality, hyperspectral imaging, sustainability

## Abstract

Seeds play a critical role in ensuring food security for the earth’s 8 billion people. There is great biodiversity in plant seed content traits worldwide. Consequently, the development of robust, rapid, and high-throughput methods is required for seed quality evaluation and acceleration of crop improvement. There has been considerable progress in the past 20 years in various non-destructive methods to uncover and understand plant seed phenomics. This review highlights recent advances in non-destructive seed phenomics techniques, including Fourier Transform near infrared (FT-NIR), Dispersive-Diode Array (DA-NIR), Single-Kernel (SKNIR), Micro-Electromechanical Systems (MEMS-NIR) spectroscopy, Hyperspectral Imaging (HSI), and Micro-Computed Tomography Imaging (micro-CT). The potential applications of NIR spectroscopy are expected to continue to rise as more seed researchers, breeders, and growers successfully adopt it as a powerful non-destructive method for seed quality phenomics. It will also discuss the advantages and limitations that need to be solved for each technique and how each method could help breeders and industry with trait identification, measurement, classification, and screening or sorting of seed nutritive traits. Finally, this review will focus on the future outlook for promoting and accelerating crop improvement and sustainability.

## 1. Introduction to Seeds, Applications, and Nutrition

A seed is the fertilized mature ovule that contains an embryo to grow a new plant via germination. Most seeds also contain endosperm, a triploid nutritive tissue rich in starch, oils, and protein to nourish the surrounding embryo [1]. As seeds are one of the foundations of human life and nutrition (70% of the human diet), seed quality traits are fundamentally important and represent a worldwide economic value of over 120 billion USD [2].

**Figure 1:**
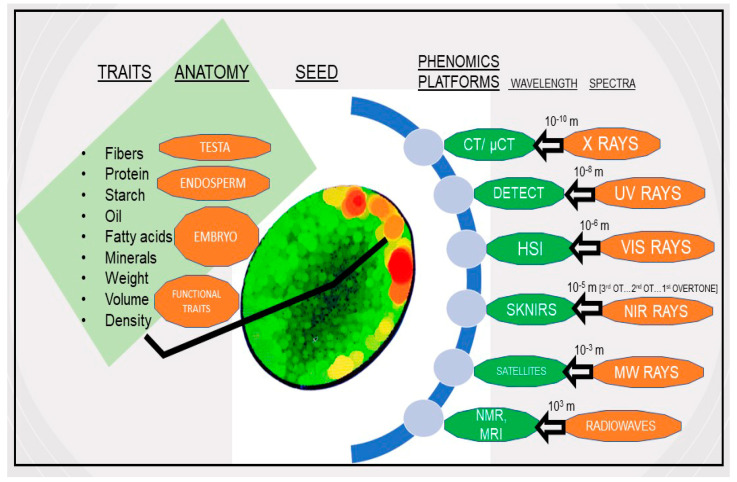
Anatomy of seeds, whole spectra modalities across wavelengths, and specific applications. NIR: near infrared; UV: ultraviolet; VIS: visible; MW: microwave; μCT: micro-computed tomography; HSI: hyperspectral imaging; SKNIRS: single kernel near infrared spectroscopy; OT: overtone; NMR: nuclear magnetic resonance; MRI: magnetic resonance imaging. Note: Some whole spectra modalities not included in the review are radiowaves and microwaves.

Seeds contain three major food compartments, including endosperm, embryo, and testa (Figure 1). Seed nutrient quality involves three major macronutrients, carbohydrates, lipids, and proteins. In addition, food crop seeds are rich in mineral nutrients such as iron (Fe), zinc (Zn), and magnesium (Mg), as well as vitamins such as vitamins E, B1, B2, and B3 [1].

Proteins are one of the three essential macronutrients. They are the building blocks of muscle cells and the skeletal bone system in humans [3]. Proteins are an essential part of a healthy and nutritious diet and are found in the endosperm of monocots and in the cotyledons of the dicots seed parts (Figure 1). Plant-based proteins can be obtained from flax seeds and legumes, including soybeans, common beans, chickpeas, and peas.

Lipids (oils and fats) are one of the essential macromolecules found in the embryo of seeds (Figure 1). Oils are one of the most important ingredients for our diets, and they can be obtained from a variety of plants, including olives, coconuts, vegetables, peanuts, flaxseed, and sesame seeds [1].

Carbohydrates are essential macronutrients because they are the vital main energy sources for humans and animals. Starch is a complex storage carbohydrate. Worldwide, starch for energy is usually obtained from staple food crops such as corn, wheat, and rice. Fibers are complex carbohydrates found in the testa and cell walls of the endosperm or cotyledon (Figure 1).

Plants and seeds also contain mineral nutritional quality traits and support human diet and health. Global seed bank awareness and mining are critical to global food security and sustainability [4]. Accurate phenotyping of mineral nutrient content and plant and seed genetic variation is, therefore, imperative for the sustainability of future food systems in the face of the world’s population increases and climate-stressed environments [5,6].

Over the past two decades, several studies have shown the diversity of seed quality traits, including through seed ionomics, the study of the elemental composition of plants and seeds [7]. Jarecki and Migut have researched legume seed quality traits and concluded that white yellow lupins were rich in protein copper (Cu), phosphorus (P), magnesium (Mg), calcium (Ca), and zinc (Zn), peas were rich in iron (Fe), faba beans were rich in P and Cu, and soybeans were rich in protein, fat, potassium (K), P, Mg, Zn, Cu, and Ca [8]. In a study with 25 faba bean genotypes, Khazaei and Vandenberg have demonstrated that low tannin faba bean genotypes had greater seed Ca, Mg, manganese (Mn), and cadmium (Cd) compared with normal tannin genotypes [9]. In a study with flax seeds, it has been reported that a serving size of 28 g of flax provided 37% of the daily value (DV) of Cu, 31% of Mn, 28% of Mg, and 19% of Zn [10].

In a pea genetic variation study, it has been reported that a serving size of 100 g of pea provided 73% of the daily value of Cu, 65% of Mn, 45% of Zn, 43% of P, 39% of Mg, 37% of Fe, 28% of K, and 8% of Ca [11]. Studies with soybeans [12] and common beans [13] have demonstrated significant correlations between Zn and other mineral nutrients. Furthermore, both studies identified and reported a core set of high nutrient content accessions for soybeans [12] and common beans [13].

## 2. Non-Destructive Seed Quality Phenomics Techniques and Platforms

Phenomics, the study of the physical and biochemical characteristics of organisms, includes the measurement of phenotypic traits, including seed protein, oil, moisture content, and morphology. Non-destructive phenomics techniques are methods used to study and measure traits without damaging the seeds or plants. There are several non-destructive phenomics techniques, including magnetic resonance imaging (MRI), fluorescence microscopy, computed tomography (CT), and spectroscopic techniques such as near infrared (NIR) spectroscopy. These techniques enable researchers to collect detailed information about the molecular, biochemical, and structural characteristics of an organism in a non-invasive way. Non-destructive phenomics techniques can be used to study the growth and development of crops and their response to climate stress and allow researchers to better understand the underlying mechanisms that drive biological processes [14].

Future food safety is an increasingly important issue, and seeds play a critical role in addressing it. Plant biologists need techniques to better utilize the broad diversity of food crop seeds. Furthermore, recent advances in non-destructive phenotyping allow data collection through rapid and cost-effective methods such as near-infrared (NIR) spectroscopy (Table 1, Figure 1).

Advances in optical sensing technologies such as hyperspectral imaging (HSI), X-ray technologies, and micro-computed tomography (micro-CT) have become valuable tools in seed phenotyping (Table 1, Figure 1). Examples of six major non-destructive seed phenomics methods are listed in Table 1.

Various detector array sensors have been used in NIR instruments. Silicon (Si) based detectors such as charged coupled devices (CCD) and photometric diode arrays (PDA) are commonly used in DA-NIR and some portable NIR spectrometers. Indium gallium arsenide (InGaAs) detectors are used in FT-NIR, SKNIR, and HIS. While InGaAs detectors cover a wide wavelength range (~905–1684 nm), Si detector technologies have a more limited wavelength range (~380–1100 nm) [15,16].

NIR spectroscopy has gathered vast attention as an alternative to wet chemistry methods in various fields for food, oil, and agriculture applications. There are various NIR spectroscopy instruments suitable for measuring products in various forms (e.g., solid powders, whole seeds, or single seeds). Overall, NIRS instruments have significant advantages, including speed, user-friendliness, and non-destructive nature. However, they require prediction or calibration model development before seed measurements can be performed (Table 1) [17].

### 2.1. Fourier Transform Near Infrared (FT-NIR) Spectroscopy (Bulk Based)

The Fourier transform near infrared (FT-NIR) spectrometer generates spectra via a Fourier transform algorithm from an interferometer system. It can be used in different modes of transmissive and reflective measurement [18] (Table 1.

FT-NIR spectroscopy has become a widely used tool; however, like any other technique, FT-NIR spectroscopy has advantages and limitations. Some of the key advantages of FT-NIR spectroscopy include high wavelength resolution, speed, and its non-invasive nature. Challenges in using FT-NIR spectroscopy are its cost and complexity (Table 1).

FT-NIR spectroscopy has been used to determine composition traits in food crops globally [19]. In a pea study [20], FT-NIR technology was used for protein prediction of whole seeds. In a study screening 840 pea samples, protein content was predicted successfully (R^2^ = 0.72). In a study with 101 maize samples from five different countries, FT-NIR spectroscopy separated their geographical origins with 98% accuracy in intact seeds [21]. A study with 152 rice varieties [22], reported that seed amylose content (R^2^ = 0.88) and fat content (R^2^ = 0.76) were successfully predicted by FT-NIR spectroscopy. Additionally, both near infrared (NIR) and mid infrared (MIR) spectroscopy were used for oilseed quantification analysis [23]. Wheat kernels and other grains were studied for seed morphology using FT-IR spectroscopy and microscopy, as reviewed by Wetzel and Brewer [24].

In a study with 20 sorghum hybrids [23], FT-NIR spectroscopy successfully predicted tannins and cellulose in both flours and whole seeds (R^2^ = 0.88). Higher prediction accuracy was reported with flours compared to non-destructed whole sorghum grains [25]. In studies with maize seeds, FT-NIR spectroscopy successfully predicted storage quality parameters such as moisture content (R^2^ = 0.96) and seed hardness (R^2^ = 0.95) [26], as well as chemical characteristics such as seed protein, fat, ash, and carbohydrates [27].

### 2.2. Dispersive Diode Array NIR (DA-NIR) Spectroscopy (Bulk Based)

Dispersive diode array NIR (DA-NIR) usually includes a dispersive grating element and a 256-diode array that collects wavelength intensities [28]. DA-NIR spectroscopy has become a widely used tool, although, like any other technique, DA-NIR spectroscopy has advantages and limitations. Some key advantages of DA-NIR spectroscopy include speed, robustness, and non-invasive nature. One challenge to the use of DA-NIR spectroscopy is low resolution (Table 1).

Seed quality traits such as protein, oil, moisture, and starch can absorb NIR light. Therefore, DA-NIR instruments can quantify seed parameter concentrations simultaneously via their unique fingerprint spectra outcome (Figure 1). DA-NIR spectroscopy instruments are more often used in on-line and at-line analysis (Table 1). In a study with soybean seeds, DA-NIR spectroscopy was used to accurately classify healthy and damaged seeds with 94% accuracy [29]. In a study with twenty-five hundred canola samples [30], DA-NIR spectroscopy successfully predicted seed moisture (R^2^ = 0.97) and oil content (R^2^ = 0.84).

### 2.3. Single Kernel NIR (SKNIR) Spectroscopy (Single-Seed Based)

**Figure 2:**
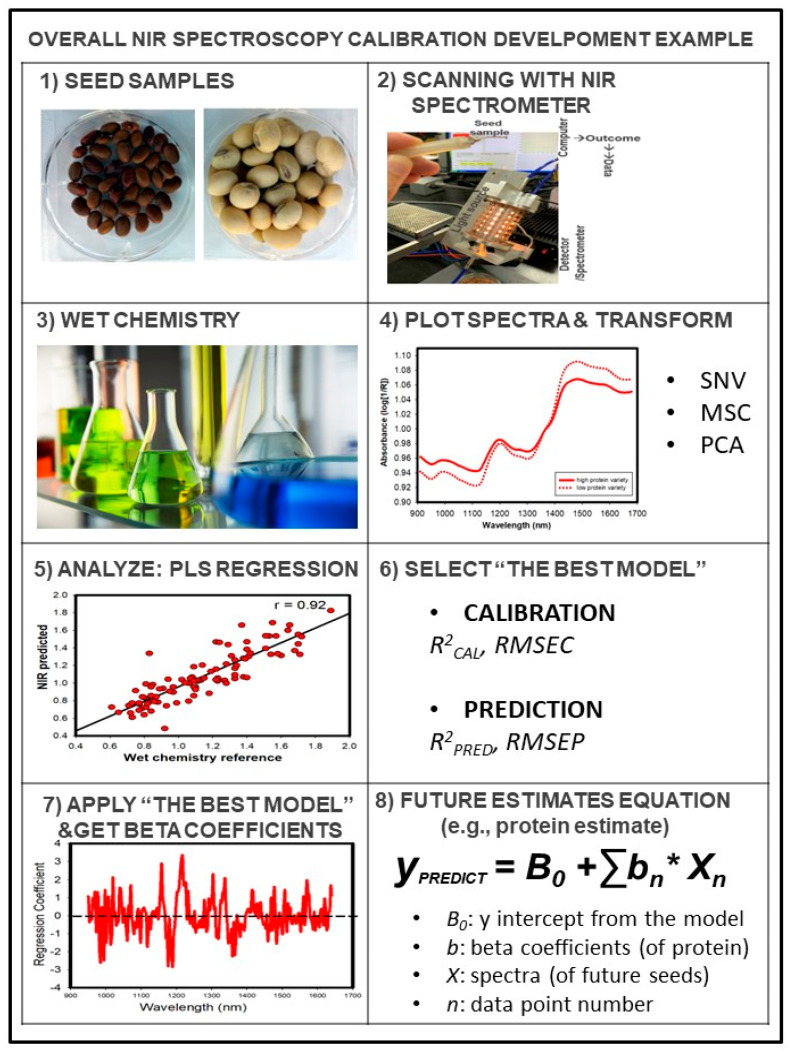
Overall calibration example for NIR Spectroscopy. After the completion of calibration and validation, calibration can be used to predict unknown seed samples for traits such as protein content. *y_PREDICT_*: prediction; *B0*: the intercept from the model; *b_n_*: beta coefficients; *X_n_*: spectra of future seeds; *n*: data point number; *RMSEC*: root mean square error of calibration; *RMSEP*: root mean square error of prediction; *SNV*: standard normal variate; *MSC*: multiplicative scatter correction; *PCA*: principal component analysis (see Section 2.3 for more details).

Recently, substantial progress has been made in single-kernel NIR (SKNIR) spectroscopy systems. SKNIR spectroscopy is not only a non-destructive and non-invasive technique, but it also enables single-seed prediction of chemical quality traits (Figure 2, Table 1).

While SKNIR spectroscopy is now widely used, it has advantages and limitations. Some of the key advantages of SKNIR spectroscopy include low cost, high speed, and its non-invasive nature. One challenge to SKNIR spectroscopy is its low sensitivity (Table 1).

SKNIR spectroscopy has been extensively used to determine composition traits, especially in the United States. Delwiche demonstrated the viability of protein predictions in single wheat seeds [31]. The Armstrong laboratory developed and tested SKNIR spectroscopy that collects spectra from single seeds while they are falling inside a glass tube [24]. More recent improvements have allowed InGaAs LED detectors to be used in faster, but more costly, SKNIRS systems [32]. Similar SKNIR spectroscopy instruments were successfully calibrated to quantify protein, seed weight, oil, moisture, and starch content in maize [33], common beans [34], soybeans [35], sorghum [36], and garden peas [17]. SKNIR spectroscopy is an indirect secondary method that requires calibration model development to relate scanned data and reference data to predict unknown seed samples (Table 1, Figure 2).

In a study with 200 varieties of wheat seeds [32], SKNIR spectroscopy successfully predicted protein concentrations based on single seeds and successfully sorted seeds for protein content [37]. In a study with single sorghum kernels [38], SKNIR spectroscopy was reported as a suitable technique for measuring the grain attributes of moisture content (R^2^ = 0.98) and kernel weight (R^2^ = 0.99), but not seed hardness. In studies with wheat and millet, Dowell et al. [39] successfully demonstrated the use of an automated SKNIR spectroscopy system and effectively sorted seeds for protein, hardness, and waxiness.

Figure 2 shows an example of calibration development for NIR spectroscopy instruments [10,33]. There are several calibration techniques for NIR instruments, including partial least squares (PLS) regression and multiple linear regression (MLR), among others [15]. A typical calibration development consists of six steps, which are described in Figure 2. The first step is to collect 50 to 100 varieties of seed samples. The second step is to scan seed samples with the NIR spectrometer and collect raw spectra data. The third step is to carry out a wet chemistry laboratory analysis for reference data. The fourth step is to pre-process the NIR spectra, including techniques such as standard normal variate (SNV). The fifth step is to develop a calibration model using PLS regression [40]. The sixth step is to obtain an equation to use for future unknown sample predictions of that specific trait [10,15,40].

*y* was defined as:

*y* = *B0* + *Σ*
*bn* × *Xn*

where *y* is the prediction, *B0* is the intercept from the model, *bn* is the regression coefficient, *Xn* is the predictor, and *n* is the sample number [40].

### 2.4. Micro Electromechanical Systems NIR (MEMS-NIR) Spectroscopy (Bulk Based)

Micro electromechanical systems NIR (MEMS-NIR) spectrometers are hand-held NIR analyzers that are used in the verification of material identity and quantification [16,41]. Some of the main advantages of MEMS-NIR spectroscopy include the ability to screen food and pharmaceuticals extremely rapidly and from anywhere (on-site measurements, Table 1). Furthermore, MEMS-NIR analyzers have the potential to be integrated into cellular phones and smartphone applications. One of the main disadvantages of MEMS-NIR spectroscopy is its limited NIR wavelength ranges (Table 1) [42]. Furthermore, Yan et al. [43] evaluated hand-held MEMS-NIR spectroscopy and reported some challenges that resulted in unrealistic promises due to the difficulty for non-experts to use the technology and interpret the data.

MEMS-NIR spectroscopy has been used in a limited number of studies to determine composition traits in plants and seeds. In a study with millet seed quality detection [44], hand-held NIR spectroscopy with a smartphone connection accurately predicted seed fat content and quality. However, in another review of miniaturization versus performance, Bec et al. [45] recommended comprehensive laboratory validation studies to adapt hand-held NIR spectroscopy technology. In a study with 110 single peanut kernels (R^2^ = 0.88) [46], a portable NIR spectroscopy predicted essential amino acids and a few non-essential amino acids with good performance. More recently, it has been reported that a newer technology with a 360° integrating sphere (GrainSense technology) could be used for handheld NIR devices for rapid measurement of grain protein, moisture, and nitrogen levels [47].

### 2.5. Hyperspectral Imaging (HSI) (Bulk Based)

Hyperspectral imaging (HSI) is an emerging non-destructive phenotyping technique that uses cameras to obtain images in multiple wavelengths (Table 1) [48]. This combination produces spectral data over spatial dimensions of imaging that can help to create seed quality content and variability maps [49]. More recently, the HSI method has been used in the determination of the chemical composition of grains [50] as well as the protein content of peanuts [51]. Moreover, HSI also has other application areas, including cultivar identification [52], crop disease identification [53], and protein detection [54]. One challenge to HSI is its very high cost and complexity (Table 1).

HSI NIR technology has been recently used for the determination of seed compositional traits. In a study with eight chickpea varieties [55], HSI-NIR spectroscopy accurately predicted single-seed protein content (R^2^ = 0.95) and was recommended for breeding studies of high-protein chickpeas. In a study with 1491 soybean samples [56], HSI-NIR spectroscopy was used to predict protein content in both seed (R^2^ = 0.90) and powder (R^2^ = 0.93) forms with high accuracy.

In a study with 35 barley varieties [57], HSI-NIR spectroscopy was able to identify barley varieties with 98% accuracy and was recommended for non-destructive seed quality and safety evaluation. In a study with 221 rice accessions, Barnaby et al. [58] concluded that HSI-NIR spectroscopy can be used for non-destructive phenotyping of rice grain chalk determination.

In a study with 100 seeds of soybean [59], HSI-NIR spectroscopy was used to successfully estimate oleic acid and linoleic acid with 93% accuracy in single seeds. It was further indicated that HSI-NIR spectroscopy has good potential for high-oleic acid soybean seed classification.

In a study with cucumber seeds [60], HSI-NIR spectroscopy accurately predicted moisture content at the single seed level (R^2^ = 0.92). Another study with 17 corn varieties [61] reported 90% accuracy in variety classification.

### 2.6. Micro Computed Tomography (Micro-CT) (Bulk or Single Seed Based)

Micro-computed tomography (micro-CT) is a high-energy radiation technique that emits X-rays to a seed sample and produces a gray image of the density of the inside seed (Table 1) [35]. Its mechanism is based on scanning rotating seed samples in 2D slices and then constructing 3D models to accurately reveal seed anatomical and morphological features, including density (R^2^ = 0.92), volume (R^2^ = 0.92), area (R^2^ = 0.92), length (R^2^ = 0.92), and width (R^2^ = 0.92) [35].

Micro-CT has become a widely used tool; however, like any other technique, micro-CT has its advantages and limitations. Some of the key advantages of micro-CT include high resolution and a non-invasive nature. One challenge to micro-CT is the very high cost and scanning times (Table 1).

**Figure 3:**
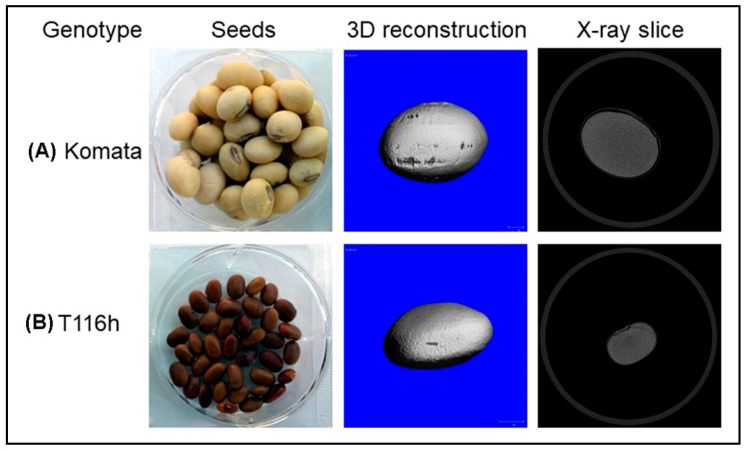
Sample images of the seeds of two contrasting soybean cultivars (**A**,**B**) after scanning with X-ray micro-computed tomography (micro-CT).

Micro-CT has been used for the determination of seed composition traits worldwide. In a study with soybeans, X-ray micro-CT successfully differentiated two contrasting cultivars and measured their density and physical properties, as shown in Figure 3. Furthermore, it has been used as a volume calculator for maize seeds [30] as well as to determine the kernel hardness of maize seeds [62].

In a study with six corn varieties [63], micro-CT successfully detected crack characteristics and breakage rate (R^2^ = 0.99) for mechanical harvesting purposes. In a study with four quinoa genotypes [64], micro-CT scanning accurately determined embryo volume from bulk seeds and seed density from single seeds.

In a study with six corn varieties [65], micro-CT accurately determined specific surface area (R^2^ = 0.93), seed volume (R^2^ = 0.58), and seed density (R^2^ = 0.93). It was further reported that the breakage rate is correlated with seed density in maize [65].

## 3. Conclusions and Future Outlook

Accurately quantifying seed functional and nutritional traits is paramount for plant breeding programs and global human nutrition sustainability [66,67,68]. Recent advances in non-destructive seed quality phenotyping are presented and discussed in this review. Among the techniques covered, SKNIR spectroscopy in seed content prediction is a rapidly maturing technology with great future potential. Its usefulness has been proven significant due to many benefits, including speed, accuracy, durability, and user-friendly operation.

In the coming decade, emphasis should be placed on more state-of-the-art and improved methodologies to develop more sensitive and less costly spectrometers together with more capable software to carry out prediction models and more robust algorithms [10,14,15,21].

To conclude, achieving more powerful and cost-effective seed quality phenomics is a critically important target for future food security and food crop breeding efforts. This article provides a review of non-destructive phenotyping methods and applications for seed biology and technology in food crop plants.

## Figures and Tables

**Table 1:** Summary of six major non-destructive seed phenomics techniques at a glance. Fourier Transform (FT-NIR), Dispersive-Diode Array (DA-NIR), Single-Kernel (SKNIR), Micro-Electromechanical Systems (MEMS-NIR), Hyperspectral Imaging (HSI), and Micro-Computed Tomography Imaging (micro-CT).

Platform	Instrument	Sample Size	Time	Other Advantages
(a) Fourier Transform (FT-NIR)	** 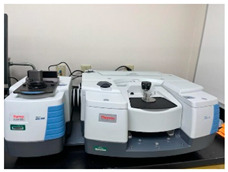 **	bulk seeds or powder	1 min	-Wavelength range: 1000–2500 nm -Benchtop device -High cost and complex -Factory calibrated (simpler calibration) -In-line and at-line measurement
(b) Dispersive-Diode Array (DA-NIR)	** 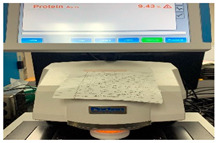 **	bulk seeds or powder	30 s	-Wavelength range: 680–2500 nm -Measures: moisture, protein, amino acids, ash, sugars, fibers -Benchtop device -Factory calibrated (simplicity) -At-line measurement
(c) Single-Kernel (SKNIR)	** 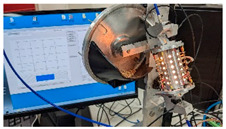 **	single seeds	300 ms	-Wavelength range: 950–1650 nm -Benchtop device -Calibration needed -Cost-effective -User-friendly, no sample prep -At-line measurement
(d) Micro-Electromechanical Systems (MEMS-NIR)	** 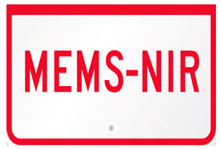 **	bulk seeds (>0.6 g) or powder	1 s	-Wavelength range: 400–1700 nm -Spectroscopy + imaging -Portable (hand-held) device -High-cost instrument -On-site measurement
(e) Hyperspectral Imaging (HSI)	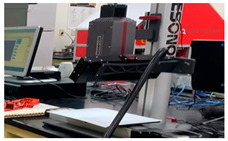	bulk seeds	6 s	-Wavelength range: 930–2500 nm -Benchtop device -Very high-cost instrument -Calibration is scanning an object with known properties (reference tile) -At-line and on-line measurement
(f) Micro-Computed Tomography Imaging (micro-CT)	** 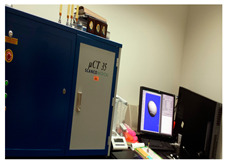 **	bulk seeds	up to 14 h	-Utilizes X-rays -Benchtop device; Time: long -Very high-cost instrument -Calibration is scanning an object with known density -At-line measurement

## References

[1] Copeland L.O., McDonald M.B. (1995). Principles of Seed Science and Technology.

[2] FAOSTAT FAO Statistical Databases. http://www.fao.org/faostat.

[3] Urry L.A., Cain M.L., Wasserman S.A., Minorsky P.V., Reece J.B. (2017). Campbell Biology.

[4] McCouch S., Baute G.J., Bradeen J., Bramel P., Bretting P.K., Buckler E., Burke J.M., Charest D., Cloutier S., Cole G. (2013). Feeding the future. Nature.

[5] Fiorani F., Schurr U. (2013). Future scenarios for plant phenotyping. Annu. Rev. Plant Biol..

[6] Dobermann A., Bruulsema T., Cakmak I., Gerard B., Majumdar K., McLaughlin M., Reidsma P., Vanlauwe B., Wollenberg L., Zhang F. (2022). Responsible plant nutrition: A new paradigm to support food system transformation. Glob. Food Secur..

[7] Salt D.E., Baxter I., Lahner B. (2008). Ionomics and the study of the plant ionome. Annu. Rev. Plant Biol..

[8] Jarecki W., Migut D. (2022). Comparison of yield and important seed quality traits of selected legume species. Agronomy.

[9] Khazaei H., Vandenberg A. (2020). Seed mineral composition and protein content of faba beans (*Vicia faba* L.) with contrasting tannin contents. Agronomy.

[10] Hacisalihoglu G., Armstrong P.R. (2022). Flax and sorghum: Multi-element contents and nutritional values within 210 varieties and potential selection for future climates to sustain food security. Plants.

[11] Hacisalihoglu G., Beisel N., Settles A.M. (2021). Characterization of pea seed nutritional value within a diverse population of P. sativum. PloS ONE.

[12] Hacisalihoglu G., Settles A.M. (2017). Quantification of seed ionome variation in 90 diverse soybean (*Glycine max*) lines. J. Plant Nutr..

[13] Hacisalihoglu G., Vallejos E. Distribution of seed mineral nutrients and their correlation in *P. vulgaris*. Proceedings of the Florida State Horticultural Society.

[14] Rahaman M.M., Chen D., Gillani Z., Klukas C., Chen M. (2015). Advanced phenotyping and phenotype data analysis for the study of plant growth and development. Front. Plant Sci..

[15] Agelet L.E., Hurburgh C.R. (2010). A tutorial on near infrared spectroscopy and its calibration. Crit. Rev. Anal. Chem..

[16] Zhu C., Fu X., Zhang J., Qin K., Wu C. (2022). Review of portable near infrared spectrometers: Current status and new techniques. J. Near Infrared Spec..

[17] Hacisalihoglu G., Freeman J., Armstrong P., Seaborn B., Porter L.D., Settles A.M., Gustin J.L. (2020). Protein, weight, and oil prediction by single-seed near-infrared spectroscopy for selection of seed quality and yield traits in pea (*Pisum sativum*). J. Sci. Food Agric..

[18] Guillen M.D., Cabo N. (2000). Some of the most significant changes in the Fourier transform infrared spectra of edible oils under oxidative conditions. J. Sci. Food Agric..

[19] Li-Chan E.C., Griffiths P.R., Chalmers J.M. (2010). Applications of Vibrational Spectroscopy in Food Science.

[20] Daba S.D., Honigs D., McGee R.J., Kiszonas A.M. (2022). Prediction of protein concentration in pea (*Pisum sativum* L.) using near-infrared spectroscopy (NIRS) systems. Foods.

[21] Schutz D., Riedl J., Achten E., Fischer M. (2022). Fourier-transform near-infrared spectroscopy as a fast screening tool for the verification of the geographical origin of grain maize (*Zea mays* L.). Food Control.

[22] Fan S., Xu Z., Cheng W., Wang W., Wang Q., Yang Y., Guo J., Zhang P., Wu Y. (2022). Establishment of non-destructive methods for the detection of amylose and fat content in single rice kernels using near-infrared spectroscopy. Agriculture.

[23] Baranska M., Schulz H., Strehle M., Popp J. (2010). Applications of vibrational spectroscopy to oilseeds analysis. Applications of Vibrational Spectroscopy in Food Science.

[24] Wetzel D., Brewer L. (2010). In situ FT-IR Microspectroscopy and Imaging of Wheat Kernels and Other Grains. Applications of Vibrational Spectroscopy in Food Science.

[25] Ejaz I., He S., Li W., Hu N., Tang C., Li S., Li M., Diallo B., Xie G., Yu K. (2021). Sorghum grains grading for food, feed, and fuel using NIR spectroscopy. Front. Plant Sci..

[26] Pandey P., Mishra G., Mishra H.N. (2018). Development of a non-destructive method for wheat physico-chemical analysis by chemometric comparison of discrete light based near infrared and Fourier transform near infrared spectroscopy. J. Food Meas. Charact..

[27] Amir R.M., Anjum F.M., Khan M.I., Khan M.R., Pasha I., Nadeem M. (2013). Application of Fourier transform infrared (FTIR) spectroscopy for the identification of wheat varieties. J. Food Sci. Technol..

[28] Armstrong P.R., Maghirang E., Xie F., Dowell F.C. (2006). Comparison of dispersive and Fourier-transform NIR instruments for measuring grain and flour attributes. Appl. Eng. Agric..

[29] Wang D., Dowell F.E., Ram M.S., Schapaugh W.T. (2003). Classification of fungal-damaged soybean seeds using near-infrared spectroscopy. Int. J. Food Prop..

[30] Sidhu H.K., Haagenson D.M., Rahman M., Wiesenborn D.P. (2014). Diode Array Near Infrared Spectrometer Calibrations for Composition Analysis of Single Plant Canola (*Brassica napus*) Seed. Appl. Eng. Agric..

[31] Delwiche S.R. (1998). Protein content of single kernels of wheat by near-infrared reflectance spectroscopy. J. Cereal Sci..

[32] Pearson T.C., Maghirang E.B., Dowell F.E. (2013). A multispectral sorting device for wheat kernels. Am. J. Agric. Sci. Technol..

[33] Gustin J.L., Jackson S., Williams C., Patel A., Armstrong P.R., Peter G.F., Settles A.M. (2013). Analysis of maize (*Zea mays*) kernel density and volume using micro-computed tomography and single-kernel near infrared spectroscopy. J. Agric. Food Chem..

[34] Hacisalihoglu G., Larbi B., Settles A.M. (2010). Near infrared reflectance spectroscopy predicts protein, starch, and seed weight in intact seeds of common bean. J. Agric. Food Chem..

[35] Hacisalihoglu G., Gustin J., Louisma J., Armstrong P., Peter G., Settles A.M. (2016). Enhanced Single seed trait predictions in soybean and robust calibration model transfer with NIR spectroscopy. J. Agric. Food Chem..

[36] Hacisalihoglu G., Armstrong P., Mendoza T., Seabourn B. (2022). Compositional analysis in sorghum (S. bicolor) NIR spectral techniques based on mean spectra from single kernels. Front. Plant Sci..

[37] Wesley I.J., Osborne B.G., Larroque O., Bekes F. (2008). Measurement of the protein composition of single wheat kernels using near infrared spectroscopy. J. Near Infrared Spectrosc..

[38] Bean S.R., Chung O.K., Tuinstra M.R., Pedersen J.F., Erpelding J. (2006). Evaluation of the single kernel characterization system (SKCS) for measurement of sorghum grain attributes. Cereal Chem..

[39] Dowell F.E., Maghirang E.B., Xie F., Lookhart G.L., Pierce R.O., Seabourn B.W., Bean S.R., Wilson J.D., Chung O.K. (2006). Predicting wheat quality characteristics and functionality using near-infrared spectroscopy. Cereal Chem..

[40] Martens H., Næs T. (1989). Multivariate Calibration.

[41] Senturia S. (2004). Programmable diffraction gratings and their uses in displays, spectroscopy, and communications. Proc. SPIE.

[42] Crocombe R. (2004). MEMS technology moves process spectroscopy into a new dimension. Spectrosc. Eur..

[43] Yan H., Han B., Siesler H.W. (2020). Handheld near-infrared spectrometers: Reality and empty promises. Spectroscopy.

[44] Wang F., Wang C., Song S. (2022). Rapid and low-cost detection of millet quality by miniature near-infrared spectroscopy and iteratively retaining informative variables. Foods.

[45] Bec K.B., Grabska J., Siesler H.W., Huck C.W. (2020). Handheld near-infrared spectrometers: Where are we heading?. NIR News.

[46] Yu H., Liu H., Erasmus S., Zhao S., Wang Q., van Ruth S.M. (2020). Rapid high-throughput determination of major components and amino acids in a single peanut kernel based on portable near-infrared spectroscopy combined with chemometrics. Ind. Crops Prod..

[47] Huber III M.R., Jankala K. (2020). Portable near-infrared spectroscopy for analysis of crops. Cereal Foods World.

[48] Thomas S., Kuska M.T., Bohnenkamp D., Brugger A., Alisaac E., Wahabzada M. (2018). Benefits of hyperspectral imaging for plant disease detection and plant protection: A technical perspective. J. Plant Dis. Prot..

[49] Manley M. (2014). Near-infrared spectroscopy and hyperspectral imaging: Non-destructive analysis of biological materials. Chem. Soc. Rev..

[50] Mahesh S., Jayas D.S., Paliwal J., White N.D.G. (2011). Identification of wheat classes at different moisture levels using near-infrared hyperspectral images of bulk samples. Sens. Instrum. Food Qual. Saf..

[51] Wang Y.J., Cheng J.H. (2018). Rapid and non-destructive prediction of protein content in peanut varieties using near-infrared hyperspectral imaging method. Grain Oil Sci. Technol..

[52] Kiani S., Van Ruth S.M., Minaei S. (2018). Hyperspectral imaging, a non-destructive technique in medicinal and aromatic plant products industry: Current status and potential future applications. Comput. Electron. Agric..

[53] Nasi R., Honkavaara E., Blomqvist M., Lyytikäinen-Saarenmaa P., Hakala T., Viljanen N. (2018). Remote sensing of bark beetle damage in urban forests at individual tree level using a novel hyperspectral camera from UAV and aircraft. Urban For. Urban Green..

[54] Caporaso N., Whitworth M.B., Fisk I.D. (2018). Near-Infrared spectroscopy and hyperspectral imaging for non-destructive quality assessment of cereal grains. Appl. Spectrosc. Rev..

[55] Saha D., Senthilkumar T., Sharma S., Singh C.B., Manickavasagan A. (2023). Application of near-infrared hyperspectral imaging coupled with chemometrics for rapid and non-destructive prediction of protein content in single chickpea seed. J. Food Compos. Anal..

[56] Aulia R., Kim Y., Amanah H.Z., Andi A.M.A., Kim H., Kim H., Lee W.H., Kim K.H., Baek J.H., Cho B.K. (2022). Non-destructive prediction of protein contents of soybean seeds using near-infrared hyperspectral imaging. Infrared Phys. Technol..

[57] Singh T., Garg N.M., Iyengar S.R.S. (2021). Non-destructive identification of barley seeds variety using near-infrared hyperspectral imaging coupled with convolutional neural network. J. Food Process. Eng..

[58] Barnaby J.Y., Huggins T.D., Lee H., McClung A.M., Pinson S.R., Oh M., Bauchan G.R., Tarpley L., Lee K., Kim M.S. (2020). Vis/NIR hyperspectral imaging distinguishes sub-population, production environment, and physicochemical properties in rice. Sci. Rep..

[59] Fu D., Zhou J., Scaboo A.M., Niu X. (2021). Non-destructive phenotyping fatty acid trait of single soybean seeds using reflective hyperspectral imagery. J. Food Process Eng..

[60] Xu Y., Zhang H., Zhang C., Wu P., Li J., Xia Y., Fan S. (2019). Rapid prediction and visualization of moisture content in single cucumber (*Cucumis sativus* L.) seed using hyperspectral imaging technology. Infrared Phys. Technol..

[61] Huang M., He C., Zhu Q., Qin J. (2016). Maize seed variety classification using the integration of spectral and image features combined with feature transformation based on hyperspectral imaging. Appl. Sci..

[62] Guelpa A., Du Plessis A., Kidd M., Manley M. (2015). Non-destructive estimation of maize (*Zea mays* L.) kernel hardness by means of an X-ray micro-computed tomography (μCT) density calibration. Food Bioprocess Technol..

[63] Peng-fei D., Xie R.Z., Wang K., Ming B., Hou P., Hou J., Xue J., Li C., Li S. (2020). Kernel crack characteristics for X-ray computed microtomography (μCT) and their relationship with the breakage rate of maize varieties. J. Integr. Agric..

[64] Gargiulo L., Grimberg A., Repo-Carrasco-Valencia R., Carlsson A.S., Mele G. (2019). Morpho-densitometric traits for quinoa (*Chenopodium quinoa* Willd.) seed phenotyping by two X-ray micro-CT scanning approaches. J. Cereal Sci..

[65] Hou J., Zhang Y., Jin X., Dong P., Guo Y., Wang K., Fan Y., Li S. (2019). Structural parameters for X-ray micro-computed tomography (μCT) and their relationship with the breakage rate of maize varieties. Plant Methods.

[66] Hacisalihoglu G., Ross Z. (2010). The Influence of Priming Treatment on Germination and Soil Emergence of Nonaged and Aged Annual Ryegrass Seeds. Seed Sci. Technol..

[67] Hacisalihoglu G. (2007). Germination Characteristics of Three Warm-Season Turfgrasses Subjected to Matriconditioning and Aging. HortTechnology.

[68] Hacisalihoglu G., Kantanka S., Miller N., Gustin J.L., Settles A.M. (2018). Modulation of early maize seedling performance via priming under sub-optimal temperatures. PLoS ONE.

